# Key challenges for the creation and maintenance of specialist protein resources

**DOI:** 10.1002/prot.24803

**Published:** 2015-04-22

**Authors:** Gemma L Holliday, Amos Bairoch, Pantelis G Bagos, Arnaud Chatonnet, David J Craik, Robert D Finn, Bernard Henrissat, David Landsman, Gerard Manning, Nozomi Nagano, Claire O’Donovan, Kim D Pruitt, Neil D Rawlings, Milton Saier, Ramanathan Sowdhamini, Michael Spedding, Narayanaswamy Srinivasan, Gert Vriend, Patricia C Babbitt, Alex Bateman

**Affiliations:** 1 Department of Bioengineering and Therapeutic Sciences, University of CaliforniaSan Francisco, California, 94158; 2 SIB—Swiss Institute of Bioinformatics, University of GenevaGeneva, Switzerland; 3 Department of Computer Science and Biomedical Informatics, University of ThessalyLamia, 35100, Greece; 4 INRA, Umr866 Dynamique Musculaire Et MétabolismeMontpellier, F-34000, France; 5 Université MontpellierMontpellier, F-34000, France; 6 Institute for Molecular Bioscience. The University of QueenslandBrisbane, Queensland, 4072, Australia; 7 European Molecular Biology Laboratory, European Bioinformatics Institute (EMBL-EBI)Wellcome Trust Genome Campus, Hinxton, Cambridge, Cb10 1SD, United Kingdom; 8 Architecture Et Fonction Des Macromolécules Biologiques, CNRS, Aix-Marseille UniversitéMarseille, 13288, France; 9 Department of Biological Sciences, King Abdulaziz UniversityJeddah, Saudi Arabia; 10 National Center for Biotechnology Information, National Library of Medicine, National Institutes of HealthBethesda, Maryland, 20892; 11 Department of Bioinformatics & Computational Biology, Genentech1 DNA Way, South San Francisco, California, 98010; 12 Computational Biology Research Center, National Institute of Advanced Industrial Science and TechnologyTokyo, 135-0064, Japan; 13 Wellcome Trust Sanger InstituteWellcome Trust Genome Campus, Hinxton, Cambridge, Cb10 1SD, United Kingdom; 14 Department of Molecular Biology, University of California at San DiegoLa Jolla, California, 92093; 15 National Centre for Biological Sciences, TIFRGKVK Campus, Bellary Road, Bangalore, 560065, India; 16 Chair NC-IUPHAR, Spedding Research Solutions SARL6 Rue Ampere, Le Vesinet, 78110, France; 17 Molecular Biophysics Unit, Indian Institute of ScienceBangalore, 560012, India; 18 Centre for Molecular and Biomolecular Informatics (CMBI), Radboud University Medical Center, Geert Grooteplein Zuid 26-28, 6525 GANijmegen, The Netherlands

**Keywords:** specialist protein resource, key challenges, biocuration, longevity, big data, misannotation

## Abstract

As the volume of data relating to proteins increases, researchers rely more and more on the analysis of published data, thus increasing the importance of good access to these data that vary from the supplemental material of individual articles, all the way to major reference databases with professional staff and long-term funding. Specialist protein resources fill an important middle ground, providing interactive web interfaces to their databases for a focused topic or family of proteins, using specialized approaches that are not feasible in the major reference databases. Many are labors of love, run by a single lab with little or no dedicated funding and there are many challenges to building and maintaining them. This perspective arose from a meeting of several specialist protein resources and major reference databases held at the Wellcome Trust Genome Campus (Cambridge, UK) on August 11 and 12, 2014. During this meeting some common key challenges involved in creating and maintaining such resources were discussed, along with various approaches to address them. In laying out these challenges, we aim to inform users about how these issues impact our resources and illustrate ways in which our working together could enhance their accuracy, currency, and overall value. Proteins 2015; 83:1005–1013. © 2015 The Authors. Proteins: Structure, Function, and Bioinformatics Published by Wiley Periodicals, Inc.

## INTRODUCTION

With the advent of the technologies of the omics age, there is far more data to manage, access, and understand than ever before. As the data are far greater than any single group of researchers can hope to ever cope with, repositories for these data are becoming increasingly important. While it is simple to say: “I have data, therefore I shall create a database for it,” there are many challenges and hurdles in doing it such that the data can be retrieved and studied effectively and efficiently. Recently, a small group of leaders of web-accessible, knowledge-based, specialist protein resources (SPRs) came together at a retreat sponsored by the Wellcome Trust to discuss the challenges they face. The retreat was held at the Wellcome Trust Genome Campus in Cambridge (UK) on August 11 and 12, 2014. Although each SPR present represented some unique challenges and issues, it became clear that there were some overarching challenges common to all of them. Together, these can be combined into a single question: What makes a database useful? Here, we discuss the top challenges that emerged from this discussion, along with some of the ways that were proposed to address them from the perspective of the researchers at the retreat. The SPRs represented at the retreat covered diverse communities, listed in Table1.

**Table 1:** The SPRs and Major Databases That Participated in the Wellcome Trust retreat, Their URLs, and Primary References

Database name	URL	Reference
*C*arbohydrate-*A*ctive *E*nz*y*mes database (CAZy)	www.cazy.org	1
ConoServer database for conopeptides	http://www.conoserver.org/	2
CyBase database of cyclic proteins	http://www.cybase.org.au/	3
ESTHER database (*EST*erases and alpha/beta-*H*ydrolase *E*nzymes and *R*elatives)	http://bioweb.ensam.inra.fr/ESTHER/general?what=index	4
ExTopoDB database of experimentally derived topological models of transmembrane proteins	http://bioinformatics.biol.uoa.gr/ExTopoDB/	5
EzCatDB database of Enzyme Catalytic Mechanisms	http://ezcatdb.cbrc.jp/EzCatDB/	6
GPCRDB (G Protein-Coupled Receptors Database)	http://www.gpcr.org/7tm/	7
gpDB (a database of GPCRs, G-proteins, Effectors and their interactions)	http://bioinformatics.biol.uoa.gr/gpDB/	8
the Histone Database	http://genome.nhgri.nih.gov/histones/	9
the IUPHAR/BPS Guide to pharmacology	http://www.guidetopharmacology.org/	10
Kinase.com	http://kinase.com/	
the KinG database (a database of protein kinases in genomes)	http://megha.garudaindia.in/king/index.jsp	11
the MACiE Database (*M*echanism, *A*nnotation and *C*lassification *i*n *E*nzymes)	http://www.ebi.ac.uk/thornton-srv/databases/MACiE	12
MEROPS (the peptidase database)	http://merops.sanger.ac.uk/	13
neXtProt (knowledge resource on human proteins)	http://www.nextprot.org/	14
OMPdb (a database of β-barrel outer membrane proteins from Gram-negative bacteria)	http://www.ompdb.org/	15
PASS2 database of structure-based sequence alignments of protein structural domain superfamilies	http://caps.ncbs.res.in/pass2/	16
Pfam	http://pfam.xfam.org/	17
Reference Sequence (RefSeq) database	http://www.ncbi.nlm.nih.gov/refseq/	18
the Structure-Function Linkage Database (SFLD)	http://sfld.rbvi.ucsf.edu/	19
Transporter Classification Database (TCDB)	http://www.tcdb.org/	20
TIGRFAMs	http://www.jcvi.org/cgi-bin/tigrfams/index.cgi	21
UniProtKB	http://www.uniprot.org/	22

## What are SPRs, and why do we need them?

The SPRs represented at the Wellcome Trust meeting are just a tiny proportion of the SPRs available to researchers, but most are designed to perform a similar function: to add value to the data available to researchers. SPRs cover a wide range of different types of protein. Some are general and relate to all types of proteins (*e.g*., Pfam); others focus on specific types of proteins, for example, transporter proteins (*e.g*., TCDB), receptor proteins (*e.g*., GPCRDB), and enzymes (*e.g*., ExCatDB). In all cases, data are available in many formats, including the primary literature and associated supplementary material, patents, and reference databases (*e.g*., RefSeq or UniProtKB). An SPR can add value to data in many different ways, from simply collating it into levels of classification, to performing complex data analysis. The most comprehensive list of SPRs can be found in the Nucleic Acids Research Database issue and its associated Molecular Biology Database Collection,^23^ published annually in January. In 2014 there were [mt]1500 databases listed in the Molecular Biology Database Collection, which range from comprehensive reference databases to resources that focus on a single protein family, and everything in between. The data types available in SPRs are just as diverse, yet there are commonalities among them. All proteins have a few features in common, namely their amino acid sequence (and often also the associated nucleic acid sequence) and the species from which they come. Thus, most SPRs will contain either a nucleic or amino acid sequence (or both), and at least a minimal amount of metadata. The data that the SPRs add, however, is myriad and varied. Some will annotate the chemistry, such as the enzymatic reaction, cofactors, regulators, and so forth. Others add three-dimensional information such as the PDB structure or active site motifs. Some add disease information, such as disease causing SNPs or polymeric forms; others look at the kinetics of the reaction and small molecule binding. If there is a study or data available in the primary literature and a group of scientists interested in that field, the chances are that there is an associated SPR. Thus, by strengthening and expanding the realm of SPRs, we can provide a richer and more diverse set of resources to the research community, and accelerate the rate at which individual results can be incorporated into interactive databases for greater use.

## Misannotation and data integrity

The foremost challenge to most SPRs is the issue of the accuracy of data and its associated annotations, not only in their own resources, but in those of others too. Many different types of error can be found in data resources, all of which present challenges for users, especially those unfamiliar with their specialized content. An analysis done in 2009 on a relatively small set of highly manually curated enzyme superfamilies^24^ showed that some major public databases misidentified an average of 5–63% across the six superfamilies studied, usually by “overannotation” of specific function when the evidence only supports annotation of general functional properties. Some errors are relatively easy to identify through automated processes and pipelines (*e.g*., MisPred,^25^ which identifies erroneous protein sequence function predictions in public databases, usually in the form of abnormal, incomplete and incorrect predictions). Others, such as errors in the underlying scientific information (*e.g*., if the protein sequence has translation errors or the biochemical characterization is incomplete) are much harder to find, especially as our knowledge continues to grow so fast that we often have to move on rather than go back to correct errors.

One example of a problem caused by the growth of knowledge is the enzymatic mechanism for lysozyme. For over 50 years the accepted mechanism involved an ion pair intermediate. It was not until 2001 that new experiments showed that the intermediate was instead a covalent glycosyl enzyme.^26^ As researchers are challenged to stay up to date with the scientific literature and SPRs to continually update their information, nonexpert users would be forgiven for thinking the ion-pair mechanism was still the definitive one (especially as this is the mechanism shown in many text books as well). Such examples raise a number of questions for our community and our users: Can we ever say that we know the correct mechanism of an enzyme? Can we ever hope to keep up with the frontier of scientific discovery? Further, even if the new information has been published, will it be incorporated into any database resources and then propagated throughout the many different SPRs? Possibly not—as the key to keeping databases up-to-date requires that curators (or users, or text mining robots) go back over the literature again and again to identify changes, new discoveries, and what information has become obsolete. Ideally, we need an exceptional solution to accurate and automated updating of all relevant databases, even including those that are deeply dependent on specialized knowledge within a field.

Another common error found in protein sequence analysis is the misannotation of a protein due to its modular (or multidomain) structure. For example, the carbohydrate-binding module (CBM) in carbohydrate-active enzymes is frequently found appended to catalytic domains belonging to various families, including domains of unknown function. A best BLAST^27^ hit matching only the CBM often leads to erroneous annotation of the adjacent domain. This is because the matched domain is often used to annotate the function of the entire protein, not just the portion found via BLAST. In many cases, such errors can only be identified when researchers go back to carefully examine specific cases in detail. For example, the aminotransferase-related enzyme (UniProtKB: B8NM72) was ultimately found to be involved in synthesis of a ribosomal peptide, rather than acting as a nonribosomal peptide synthetase as previously thought.^28^ Such annotation transfer errors can often lead researchers astray and highlights why expert manual annotation is so essential for SPRs.

Although over-prediction, transferring annotation from one annotated protein to another of unknown function using relatively lax parameters, has the advantage of increased data coverage, it can lead to many erroneous function predictions. Such annotation errors can be further compounded by “proof by repetition”; the assumption that the most numerous annotations are the correct ones.^29^ Such errors can be protected against by “under annotation,” that is, transferring data only when we have the highest confidence that it is accurate, for example, in requiring not only a high confidence BLAST score, but also in having the active site profile fully matched. These protocols often lead to significantly fewer annotations being assigned, but the quality of the annotation transfer is much better. In both cases, annotation transfer is further complicated by the fact that a protein’s function can be defined as the molecular/chemical role (*e.g*., a specific serine kinase) or the broad biological process the protein mediates (*e.g*., mediating the coagulation of blood). Generally, it is quite difficult to decipher the biological role of a protein in the physiological context using computational methods and therefore such predictions should be used with caution. Nowadays a BLAST search of the nonredundant protein database of NCBI (RefSeq) or on UniProtKB often identifies a large number of similar proteins originating almost exclusively from genome sequencing (that is, these proteins have had no characterization performed). Close examination of the names attributed to these proteins shows that they are both heterogeneous and transmitted from one to another via automated processes (creating a mess that is increasingly difficult to discern and fix).

Many protein homologues lack one or more critical residues, making them functionally inactive, another aspect of annotation transfer that may lead to erroneous annotation. These proteins may be biologically relevant but with another function, or on the other hand, the missing residues could be artefacts caused by gene assembly errors. Other typical gene assembly errors lead to the prediction of putative proteins where the wrong initiating methionine has been identified, or where exons have been omitted. Although such errors may be subsequently corrected, finding the time to back-check for these types of errors requires more resources than are available to many SPR curators.

## Fixing annotation errors and propagating data

Once an error is identified, how do we fix it? Many resources, such as UniProtKB and RefSeq, have mechanisms for users to report problems so that annotation errors can be corrected. Additionally, specialized resources have been developed to help address this issue and provide at least some reannotations (such as PDB_REDO^30^ for PDB atomic coordinates). However, many others, such as the Protein Data Bank (PDB),^31^ GenBank,^32^ the European Nucleotide Archive (ENA),^33^ and the DNA Data Bank of Japan (DDBJ),^34^ lack these procedures as they are primary repositories that are designed to archive original data. SPRs tend to have their own policies for correcting errors that are relevant to the specific nature of each resource. Many welcome (and need) input from expert users in order to identify and correct data errors.

Identifying the error is only the first step. Once we know an error exists, how do we propagate the fix through all the SPRs that utilize the original entry? The provenance of a datum is often difficult to identify. Have the database curators taken information directly from UniProtKB, or RefSeq, or from the primary literature? Maybe they took it from another resource, but where did that resource’s curators get it from? While such repurposing of data is common place, it is a good way to propagate annotation errors. A better solution might be for all primary data to be stored in a common archive resource so that niche or derived databases could provide pointers to the original information that could then be expanded on demand. However, such an annotation archive would be challenging to implement on a wide scale.

Another promising approach used by some resources (*e.g*., the Gene Ontology (GO),^35^ UniProtKB, and some SPRs) is to use the concept of “evidence,” sometimes in combination with the use of the Evidence Code Ontology (ECO),^36^ a structured and controlled vocabulary for evidence in biological research. Used in the context of protein function annotation, evidence codes allow for evidence not only to have a type (*e.g*., inferred from electronic annotation), but to have a source (*e.g*., a specific resource), providing users with an effective way to judge the confidence with which to judge an annotation. Other SPRs are starting to follow suit although populating a resource with this sort of “back annotation” can be a long and often complicated process as all the data must be cross-checked and back-edited. That being said, if we are ever to propagate annotation “fixes,” the ability to follow data back to their source is going to be critical, suggesting the value of using ECO (or something similar) moving forward.

With the ever increasing volume of data, how do we grow and maintain our resources responsibly, especially in the context of the misannotation challenge? Specialist curation (by individuals who are highly trained to a particular resource and/or in a particular field) is always going to be critical because databases that include a high degree of cross-check and human curation provide significant added value over simple repositories or meta-resources/hubs that are especially prone to propagation of misannotation. For example, the IUPHAR/BPS Guide to PHARMACOLOGY uses expert curators for particular protein “receptor” types that are linked to subcommittees of experts who ensure data quality. This approach allows experts to keep their field “clean” and to benefit from highly cited publications^37^ rather than using “data trawling” which can lead to misleading information being propagated.

## The user’s role in expanding SPR data coverage

Users of the SPRs are going to become increasingly important to correcting errors and growing SPRs in the future. For example, expert users are in a position to inform SPRs of errors that they have spotted or to contribute new entries based on their experiments and/or publications. Depending on an evaluation of the evidence provided, the resource can then update the entry. In the experience of the attendees at the retreat, the major hurdle to adopting user based annotation methods is educating users about the benefits of contributing their information to database resources versus the effort of creating the annotation themselves (commonly referred to as the “tragedy of the commons”^38^). One route to achieving more user input (such as that taken by the international crystallographic community) is to require data entry before the results can be published. Without the support and enforcement by the journals, however, it is not practicable to capture functional information efficiently in this manner. On the bright side, the level of detail repositories require of their depositors need not be onerous. For example, including the EC number along with a sequence accession number for an enzyme would represent enormous progress that would allow SPRs and larger resources alike to incorporate research results keyed to those common identifiers. The flip side of encouraging annotation and error correction contributions by users is that the manual incorporation of this information could quickly outgrow a resource’s ability to keep up. Again, user submissions enabled via structured information formats supported by the journals would offer progress toward more automated solutions. The International Society for Biocuration (http://www.biocurator.org) is an active proponent in bringing scientists, curators and journals together with a view to enable user submissions. The annual International Biocuration Conference is a great opportunity for these groups come together to discuss the challenges involved.

A different route to maintaining data quality is the use of the Wikipedia model. Rfam^39^ and Pfam^17^ both utilize this model to populate the respective databases with Wikipedia pages created by authors. Although both of these approaches are promising, general application of this model awaits answers to several basic questions: which resources become the primary repositories of user-contributed data? How do we deal with overlapping resources? When an old resource is retired, who takes on its data? Will one resource become the ultimate one for all protein annotations, which are then used and elaborated upon by SPRs? Will all journals agree to the process? Will the annotation process be both simple and complete enough that authors find benefit to the process? There are no simple answers to these questions, but as the amount of data grows, many aspects of protein research would benefit if SPR developers, users and publishers begin to work together in developing a common plan for moving forward.

## Weathering the data deluge

Even the best resource must keep its information up-to-date, and in this omics era possibly the biggest challenge we face is the sheer volume of data currently available, along with its projected growth at a near exponential rate. There is also a constant growth in the number of data-sources. UniProtKB and RefSeq have approximately 89 million and 47 million entries, respectively, as of November 2014, of which just over half a million are manually annotated or reviewed in each database and over 70% of these reviewed proteins are annotated via similarity to a protein of known function. For every bit of information on a single protein that exists, there are even more proteins for which we have no data, save a primary amino acid sequence. SPRs have two important roles to play in weathering this data deluge: one is to provide novel annotation and understanding in their field of expertise and the other is to provide online tools to access those annotations. Additionally, SPRs must determine what information to submit to larger and more general resources, and which to glean from other data resources. These roles, in turn, aid resources such as RefSeq and UniProtKB in extending and improving their predicted annotations. A good SPR should know where its strengths lie and clearly distinguish the primary annotations for which they are the unique source from those data that come from other resources.

## Adoption of best practices

Along with annotation input from experimental users, the Retreat discussion also suggested that the SPRs could benefit from best practices that have been developed. However, for any one resource to be useful to another, the language that they both use needs to be standardized. One such exercise in standardization was the EMBRACE project (http://www.embracegrid.info/40) which worked to integrate major databases and software tools in bioinformatics, using existing methods and emerging Grid service technologies. Some resources already use the same language, conceptually facilitating the exchange of information between them. For instance, OMPdb uses the commonly accepted family classification system of Pfam. But generally, what one resource means by the term «family» or «superfamily» might not be what another means. For example, the SFLD definition requires that the proteins not only be evolutionarily related, but that they have a conserved chemical aspect to their function. TIGRFAM, on the other hand, only requires evolutionary relatedness.

While we are not advocating that all resources use an identical language (biology is nothing if not messy, so a term in one field will not directly translate to another), there needs to be a way to both establish and translate concepts. Ontologies are certainly the most robust method to do this, and SPRs need to define their language and concepts clearly so that mapping is possible between the different SPRs. Although the Gene Ontology (GO) is probably the most widely known ontology in the field of bioinformatics, a PubMed search for “ontology” in the title of a article yields almost 1500 hits (almost 500 of which involve GO). Especially for some key concepts of biochemistry and biology, relevant to SPRs, the capability exists to link data across resources that share similar data. The ontology repositories, such as BioPortal^41^ and the OBO Foundry,^42^ offer a good way to find an ontology that will help describe specific types of data by collecting as many biological ontologies as possible into a single location. At the very least, an understanding of the terms used by various resources will allow SPRs to map data between one another, benefitting both our curators and users.

## Resource longevity

The 2014 Nucleic Acids Research database issue contained 58 new databases and updates to 123 existing databases, growing the total number of databases represented in the online collection of molecular biology databases (http://www.oxfordjournals.org/our_journals/nar/database/c/) to 1552 in 2014.^23^ It is quite easy to create a database, and many databases are created as part of a PhD or Masters projects, but with no plan for future maintenance of the information. Even for more established SPRs, it remains hard to maintain such resources over many years. A 2008 study found that almost 40% of database URLs published in journals were no longer regularly available.^43^ “Zombie databases” are not maintained past the original publication for reasons that range from lack of interest or funding to a career move by the creator. Over time, these may become unreliable or even misleading by failing to keep current with the field, including naming conventions, links to other databases, or even browser compatibility, and eventually are taken down. One answer to the problem of longevity is greater integration. An example of such an approach is InterPro,^44^ a resource that integrates eleven different protein domain and family resources into a one-stop-shop. The member databases still retain their own identity, data, and role in the wider community while InterPro provides access to their annotations (and expertise) through a single website. The caveat to the inclusion of a new SPR within InterPro is that the source database must have sequence analysis methods that are reproducible and scalable, so is unlikely to be suitable for all SPRs, for example, CAZy, where sequences are annotated on an individual basis. One of the roles performed by InterPro is the provision of the annotations produced by its member databases to UniProtKB on a monthly database, such that annotations are up-to-date with the source member database and all sequences found in UniProtKB. The advantage to the InterPro user is the ability to view all the different annotations in a single resource. To be able to view broad- and fine-grained annotation in a single interface is highly efficient, so in this respect it is arguable that the whole (InterPro) is greater than the sum of its parts (member databases).

Similarly, Pfam (which can be considered both a SPR and a reference database) uses the annotations found in many of the SPRs as either starting points for generating new entries and/or for annotating existing Pfam entries. For example, most of the peptidase families in Pfam have been derived from or annotated using MEROPS (note that Pfam does not contain any of the fine-grained subfamily annotations found in MEROPS). In many ways SPR data integration into Pfam parallels that of data integration of member databases into InterPro; however, the attribution to the source SPR is less obvious than with InterPro. Also, Pfam will derive its own profile hidden Markov model for the entry and possibly supplement the SPR annotation. There is also the risk that smaller SPRs will be subsumed by Pfam and in so doing will reduce traffic to the individual resources. Furthermore, as annotations of both proteins and domains are updated in both in the literature and SPRs, there is no rigorous mechanism in the Pfam production software pipelines to identify and reconcile the differences between Pfam and the SPRs. However, a major advantage of Pfam is its wide use within the scientific community. Moreover, it is a founder member database of InterPro and is used within CDD.^45^ Thus, the information in Pfam, both curated by Pfam and derived from the SPRs, is propagated to a broad audience.

There are several other examples of consortia that work toward greater integration of protein resources, and although not formally represented at the inaugural SPR meeting, these have proven to be exceedingly useful. Two such examples are the HUPO Proteomics Standards Initiative^46^ and the International Molecular Exchange (IMEx) Consortium of Protein-Protein Interaction databases.^47^ The IMEx Consortium is an excellent example of where coordination of a set of related databases has led to standardization and improved interoperability. (UniProtKB was the only member of this consortium represented at the SPR meeting.)

For many small SPRs attending our retreat, a continuing challenge to longevity is obtaining funding. In contrast, this is not a consideration for the Histone Database and the RefSeq Database as these are supported by intramural funds at the National Institutes of Health. Several models currently exist for funding SPRs. These include: Self-funding, that is, they are maintained using funds provided to the research group by the board of directors of the home institute for normal running of the group, for example, GPCRDB and DSSP,^48^ grant agency funding, for example, the SFLD is currently supported by NIH and NSF grants, user-based funding (commercial), for example, KEGG,^49^ which, due to lack of other funding resources, is now forced to operate via paid licensing fees, and user base “funding” (public), for example, the Little Skate Genome Project,^50^ which held many jamborees to annotate the skate genome, minimizing the need for a large curator and bioinformatics staff employed by the resource. It is our job, as a community of SPRs to help one another and to listen to our users. Furthermore, those of us in the SPR community need to work together to minimize duplication of effort, helping one another to maintain quality as well as quantity so that our users have the best possible data from which to work. We also need help from our user communities, without whom our resources cannot hope to thrive. Finally, as funding for the Wellcome Trust Retreat was of necessity limited to a small group of database resources, we would like to encourage any researchers that run their own SPRs to join our mailing list (https://listserver.ebi.ac.uk/mailman/listinfo/sprn) and contribute to further discussion about the issues described in this brief report, including greater data interoperability, standardization, and consolidation.

## CONCLUSIONS

SPRs are critical to biological research in this omics age and we can all benefit from sharing the many different technical approaches that have been developed. This is especially true for meeting the challenge of creating user-friendly web interfaces and supporting complex queries, both of which are difficult to develop and maintain. Such technical developments represent a high burden on many SPRs and their requirements can quickly grow beyond an SPR’s interest and expertise. Still, many of us have developed similar data structures which could allow us to share methods for developing web interfaces and adoption of external tools. A further useful extension would be an agreement among SPRs to make their data available to other resources in some fairly standard or well defined format, attended by assurance of the quality and provenance of the contributed data/annotations (*e.g*., when the data were last updated and where they came from). Finally, greater coordination and collaboration between databases is going to be even more important with the ever growing amount of data available. Here, a synergy between SPRs and reference databases will be essential, with the SPRs improving the accuracy and quality of their data and the reference databases integrating these data for broader dissemination to the research community.
